# Sensor behavior of MoS_2_ field-effect transistor with light injection toward chemical recognition†

**DOI:** 10.1039/d1ra03698j

**Published:** 2021-08-03

**Authors:** Md Iftekharul Alam, Tsuyoshi Takaoka, Hiroki Waizumi, Yudai Tanaka, Muhammad Shamim Al Mamun, Atsushi Ando, Tadahiro Komeda

**Affiliations:** Department of Chemistry, Graduate School of Science, Tohoku University 6-3, Aramaki-Aza-Aoba, Aoba-Ku Sendai 980-8578 Japan; Institute of Multidisciplinary Research for Advanced Materials (IMRAM, Tagen), Tohoku University 2-1-1, Katahira, Aoba-Ku Sendai 980-0877 Japan tsuyoshi.takaoka.b1@tohoku.ac.jp tadahiro.komeda.a1@tohoku.ac.jp; Device Technology Research Institute, National Institute of Advanced Industrial Science and Technology (AIST) 1-1-1 Umezono Tsukuba Ibaraki 305-8568 Japan

## Abstract

The application of field-effect transistor (FET) devices with atomically thin channels as sensors has attracted significant attention, where the adsorption of atoms/molecules on the channels can be detected by the change in the properties of FET. Thus, to further enhance the chemical sensitivity of FETs, we developed a method to distinguish the chemical properties of adsorbates from the electric behavior of FET devices. Herein, we explored the variation in the FET properties of an MoS_2_-FET upon visible light injection and the effect of molecule adsorption for chemical recognition. By injecting light, the drain current (*I*_d_) increased from the light-off state, which is defined as (Δ*I*_d_)_ph_. We examined this effect using CuPc molecules deposited on the channel. The (Δ*I*_d_)_ph_*vs.* wavelength continuous spectrum in the visible region showed a peak at the energy for the excitation from the highest occupied orbital (HOMO) to the molecule-induced state (MIS). The energy position and the intensity of this feature showed a sensitive variation with the adsorption of the CuPc molecule and are in good agreement with previously reported photo-absorption spectroscopy data, indicating that this technique can be employed for chemical recognition.

## Abstract 2

The application of field-effect transistor (FET) devices with atomically thin channels as sensors has attracted significant attention. We further explore the method to attach the chemical recognition capability by combining with light injection.

## Graphical abstract

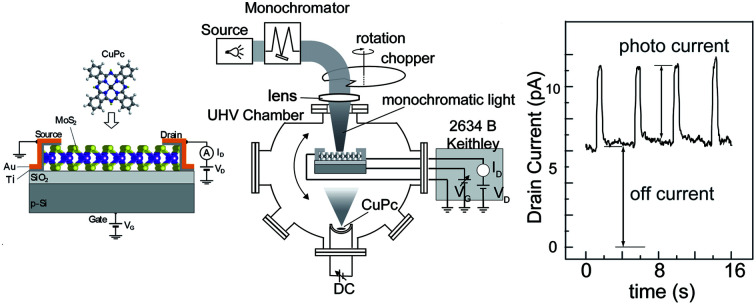

## Introduction

There is a great demand for miniaturized sensors, which enable the on-chip integration of sensors, and analyses/processes can be applied for their operation under *in vivo* conditions.^1–3^ For such an application, the field-effect transistor (FET) sensor using atomically thin channel can be a promising candidate. Due to the large area-to-volume ratio of the thin channel layer, the atomic-layer FET can exhibit higher sensitivity for the adsorption of an atom/molecule on its channel than that the conventional MOS-FET sensor device. Schedin *et al.*^4^ demonstrated that a FET device with a graphene channel works as a molecule sensor. Particularly, due to the metallic nature of graphene in its natural state,^5^ there is intensive research for alternative materials that can be readily used as the FET channel.^6,7^ It was demonstrated that mechanically cleaved MoS_2_ works as a FET channel material without further miniaturization, and MoS_2_-FET is a good sensor device.^8^ It was shown that this type of device has high sensitivity,^9^ and the detection limit can be as small as 0.8 ppm for NO gas.^10^

For these sensor devices, the adsorption of a molecule on their channel is detected by the measurement of the drain current change, which is caused by the charge transfer or the polarization of the molecule. However, this technique has no significant chemical sensitivity for the adsorbate. Several studies demonstrated the chemical sensitivity of FET sensors using a host–guest-type reaction on the channel, where the formation of chemical bonds between the pre-adsorbed receptor on the channel and the target molecule changes the FET property.^11–14^ However, it is not practical to prepare receptors for various types of adsorbates, especially for the establishment of general purpose sensors.

Thus, to realize a sensor with chemical sensitivity, methods to make the chemical property of the target molecule manifest in the FET property have to be developed. Accordingly, it may be intriguing to explore how the FET property changes when a molecule on the channel is exposed to light with specific wavelengths. The ultraviolet-visible (UV-vis) absorption spectroscopy of the interface between molecules and an atomically-thin MoS_2_ surface revealed many intriguing electronic features such as the HOMO–LUMO excitation energy, molecule-induced interface feature and dissipation dynamics of the excited electrons. However, it has not been reported to date whether these features of adsorbates and the interface can be revealed from the electrical properties of FET. To judge this capability of FETs, spectroscopic measurement of the FET property upon the injection of monochromatized light onto the channel is required. If this type of behavior is confirmed for an FET device with an MoS_2_ channel, it is possible to endow chemical sensitivity to the MoS_2_-FET sensor.

Herein, we examined this effect of coper phthalocyanine (CuPc) deposited on the channel of an FET device and the FET properties were measured, focusing on the photo-induced drain current *vs. λ* spectra. Even though the pioneering work by Pak and coworkers revealed the variation of in photoresponsivity with the deposition of CuPc molecules, little is known about the spectroscopic behavior of a molecule-adsorbed FET with light injection. Upon the adsorption of the CuPc molecules, we observed the appearance of peak in the (Δ*I*_d_)_ph_*vs. λ* spectrum, the energy of which corresponds to the excitation from the highest occupied molecule orbital (HOMO) to the molecule-induced state (MIS) near the CBM of MoS_2_. This molecule-induced feature showed a sensitive variation with a small amount of molecules deposited, whose behavior showed good agreement with the photo-absorption spectroscopic data of HOMO–MIS excitation. The results indicate that the light-irradiated MoS_2_-FET sensor proposed herein has chemical recognition capability.

## Experimental

**Fig. 1:**
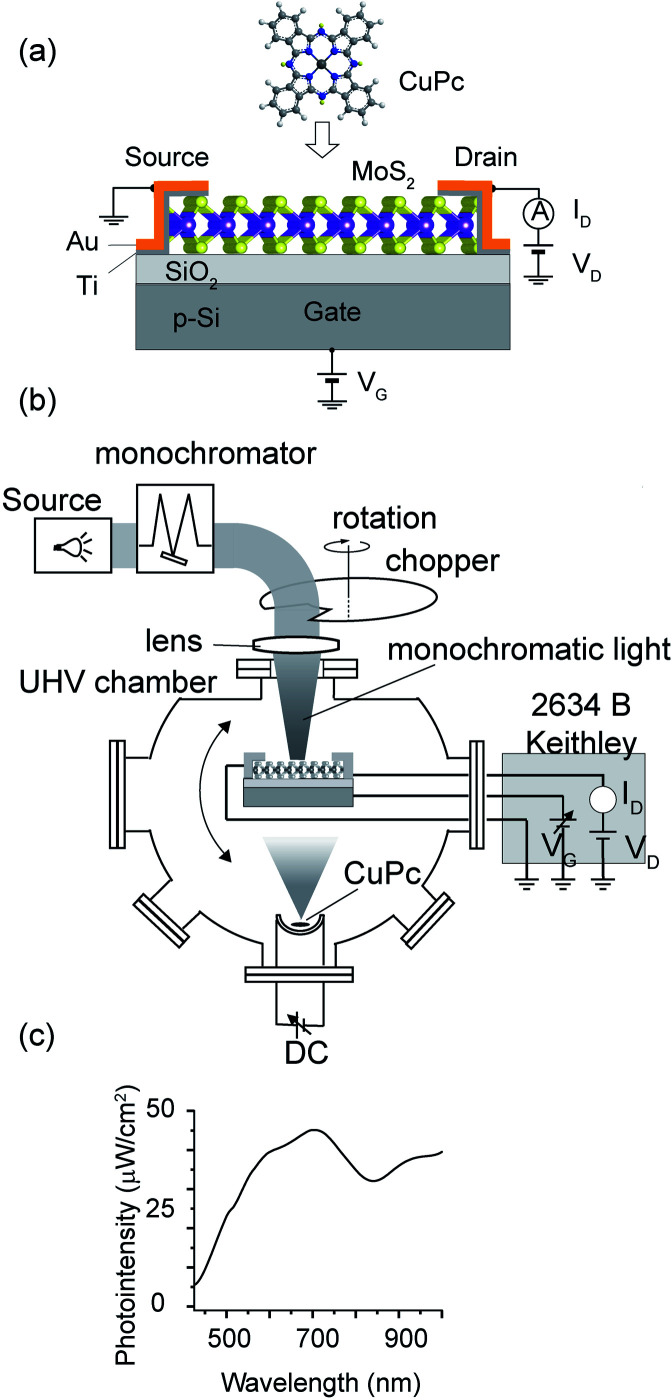
Schematic of the FET with MoS_2_ channel and molecule deposition (CuPc) (a) experimental apparatus contained in an ultra-high vacuum (UHV) chamber (b). (c) Intensity of light on the sample as a function of wavelength.

In the experiment, we fabricated the MoS_2_-FET device and examined its electronic properties of the pristine MoS_2_ channel and after the deposition of a molecule on the channel. The FET device is illustrated in Fig. 1(a). MoS_2_ flakes were exfoliated from a natural crystal using the adhesive tape method on a 285 nm SiO_2_ layer formed on a heavily doped p-type Si substrate. This SiO_2_ thickness was chosen for the optical determination of the layer number of the MoS_2_ flakes by examining the color contrast. For all the devices used in this experiment, the layer number was estimated to be three monolayers. The MoS_2_ channel was flanked by the source and drain electrodes, where the exposed channel area had the size of 1 × 1 μm^2^. The back-gate voltage, *V*_g_, was applied through p++-Si. The source and drain electrodes were formed *via* the combination of 5 nm-thick Ti and 100 nm-thick Au deposited by electron-beam lithography and DC sputtering.

We utilized ultra-high vacuum (UHV) conditions, the details of which are mentioned elsewhere.^15,16^ The MoS_2_-FET was installed inside the UHV chamber (∼10^−7^ Pa) equipped with the required shielded wirings and bonding, in which the MoS_2_ channel was cleaned by heating the sample at 150 °C, and molecule deposition and FET characterization were done *in situ* without exposing the device to the air.

For the injection of light, we used light emitted from a tungsten–halogen lamp (ASBN series, Spectral Products), which was monochromatized (CM110, Spectral Products) and guided by an optical fiber. The light injection time was controlled by a rotating chopper, as illustrated in Fig. 1(b). The chopper rotated with a periodicity of 4 s, and the dwell time of the light was set as 0.5 s in each cycle. The light was injected on the channel part of the FET using a focusing lens. The lens was positioned close to the viewport of the vacuum chamber. The intensity of the monochromatized light was measured at the sample position using a power meter (FieldMaxII-TO, Coherent) with a semiconductor sensor (OP-2 VIS, Coherent). The MoS_2_ channel part had a length of 2.79 μm and width of 2.47 μm.

Detailed information about the preparation of the device for vacuum measurement and the light experiment is presented in the ESI (Fig. S1 and S2†), respectively.

## Results and discussion

**Fig. 2:**
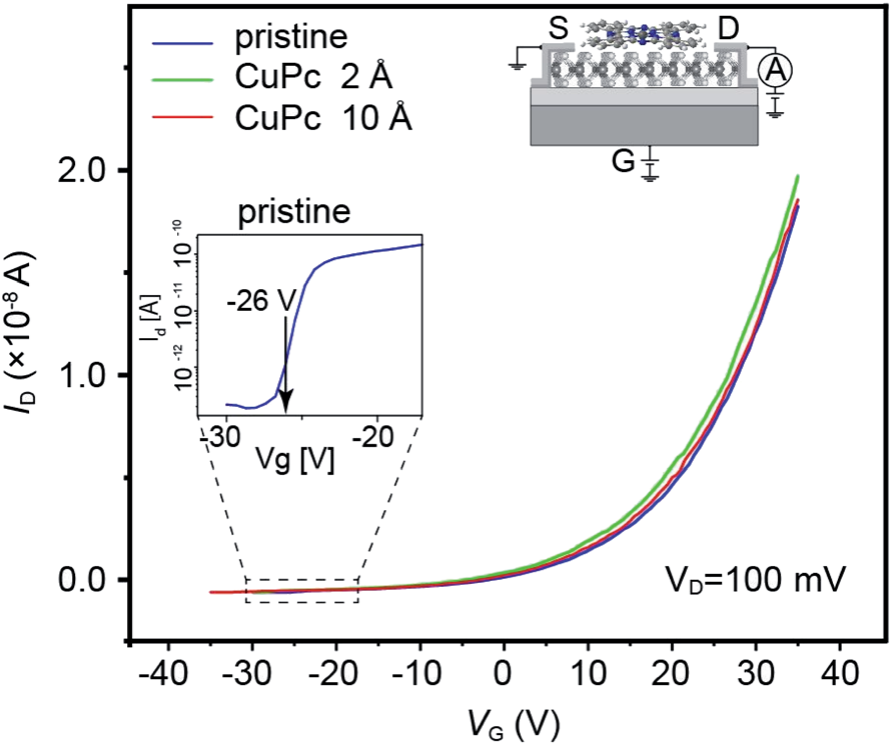
Transfer characteristics shown as *I*_d_*vs. V*_G_ plot for the channel conditions of pristine (blue), 2 Å CuPc (green) and 10 Å CuPc (red) deposited samples.

The MoS_2_-based FET used in this experiment showed the transfer characteristics illustrated as the blue solid line in Fig. 2, which showed the typical n-type semiconductor behavior. The *I*_d_–*V*_g_ characteristics after the deposition of CuPc molecules with a thickness of 2 and 10 Å are shown as plots with green and red colors, respectively. The estimated threshold voltages for the pristine, 2 Å CuPc and 10 Å CuPc deposited surfaces from the spectra shown in Fig. 2 are 18.8, 18.7 and 19.1 V, respectively. The magnitude of the shift is smaller than that reported in the previous study by Pak and coworkers,^17,18^ where the threshold voltage shifted to the positive gate voltage direction when the deposited CuPc thickness increased from 1 nm to 30 nm. The reasons for this discrepancy can be considered as follows: (1) the difference in the examined thickness of CuPc, where the maximum thickness in this work was 1 nm and (2) the treatment of the channel surface, where we annealed the MoS_2_ channel under UHV condition before the deposition of CuPc *in situ*, followed by measurement in the same chamber. In the case of the second reason, we previously reported that a large difference appeared between the *I*_d_–*V*_g_ curves obtained in air and under UHV condition.^15^ Thus, this interface difference may also appear in the *V*_th_ behavior.

**Fig. 3:**
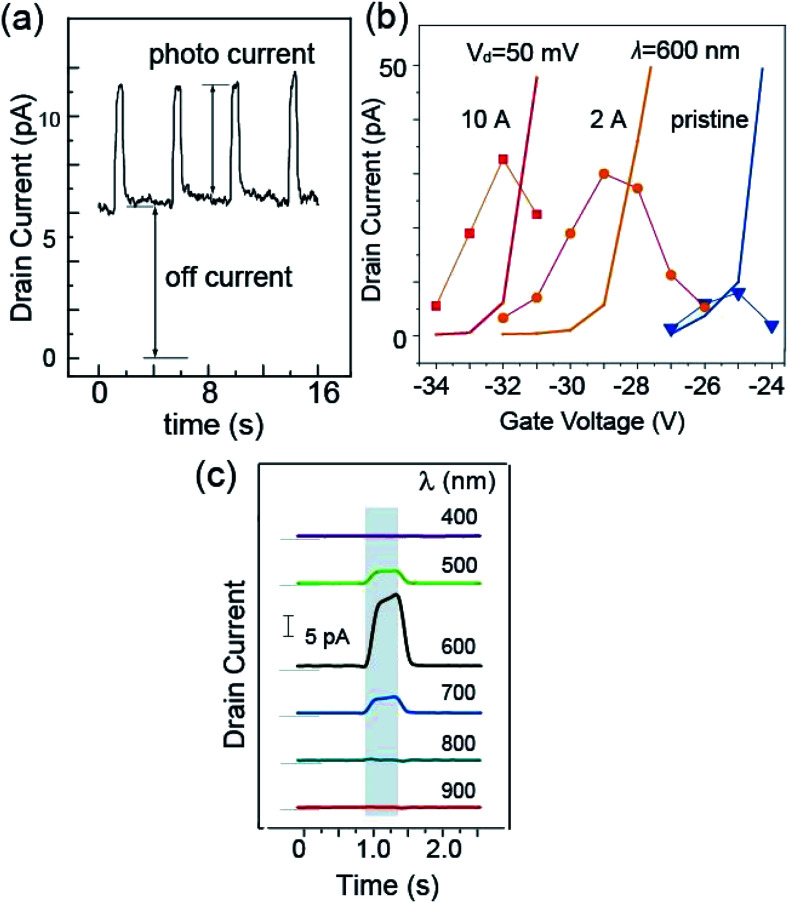
(a) Variation in drain current with the injection of 600 nm light for 0.5 s at 4 s intervals (drain voltage of 100 mV). An increase in the drain current can be seen during photoirradiation. (b) Variation in photocurrent (dots) and off-current (solid-line) with gate voltage (irradiation of 600 nm light) for the pristine (blue), 2 Å CuPc-deposited (orange) channel and 10 Å CuPc-deposited (red) channel. (c) Variation in photocurrent with the injection of light with different wavelengths ranging from 400 to 1000 nm after deposition of 2 Å CuPc.

Upon the injection of the light, a change in *I*_d_ was observed, as is illustrated in Fig. 3(a). For this measurement, we used light with a wavelength of 600 nm. Using an optical chopper, we repeated the cycle of illumination of 0.5 s followed by 3.5 s dark condition. As can be seen clearly in Fig. 3(a), *I*_d_ was enhanced during light illumination. For further analysis, we denoted the current value when the light was off as the off-current and the increase in current from the off-current when light was injected as the photocurrent, (Δ*I*_d_)_ph_.

Considering the photocurrent enhancement with the deposition of CuPc, we needed to focus on the shift in the *I*_d_–*V*_g_ curve due to the doping effect of the molecule adsorption. Thus, we first investigated the variation in the photocurrent and off-current with *V*_g_. In Fig. 3(b) we plotted the variation of these two values with *V*_g_, where the photo-current is indicated by dots and the off-current is shown as solid lines. We examined these values with three conditions of the channel, namely, pristine, 2 Å CuPc adsorption, and 10 Å CuPc adsorption, which are colored blue, orange and red, respectively.

*I*_d_ increased rapidly with *V*_g_. The *V*_g_ value at which *I*_d_ reaches 10 pA (named (*V*_g_)_r_) was found to be −25.0 V, −29.0 V and −32.0 V for the pristine, 2 Å CuPc-deposited, and 10 Å CuPc-deposited channels, respectively. The shift in (*V*_g_)_r_ can be attributed to the electron doping effect by the deposition of the CuPc molecule onto the channel with a thickness in the range of 0 to 10 Å.

Interestingly, the photo-current showed the maximum around (*V*_g_)_r_ in all cases. It should be noted that the photo-current was not calibrated with the off-current and the raw photo-current showed the maximum at around (*V*_g_)_r_. The mechanism for the appearance of the photo-current maximum with *V*_g_ will be discussed later. If the maximum photo-current of the pristine and 2 Å CuPc cases is compared, it is 3.5 times higher in the latter case. It is apparent that the photo-current was enhanced in the presence of the CuPc molecules on the channel. Thus, in the subsequent experiments, we used this *V*_g_ for the ratio of the photo-current and the off-current to be the maximum, providing the best signal-to-noise ratio.

(Δ*I*_d_)_ph_ showed a large dependence on the wavelength of the injected light. Fig. 3(c) shows the plots of the photo-current, where the channel was covered with 2 Å CuPc molecules and the light with a selected wavelength was injected. The enhancement in the photo-current can be seen in the shaded area of the plot, during which the light was injected. The base of *I*_d_ is illustrated by the thin line on the left hand side of each curve. It is obvious that the photo-current was strongly enhanced upon exposure to light with a wavelength of 600 nm.

**Fig. 4:**
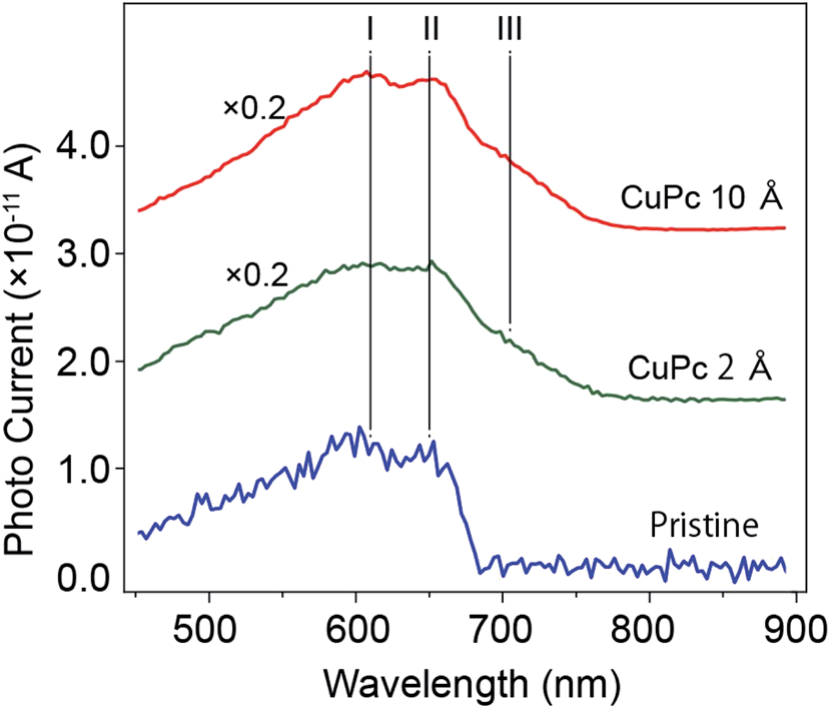
Plot of the photo-current as a function of the wavelength of the incident light for the channels of the pristine MoS_2_, 2 Å and 10 Å CuPc-deposited conditions. *V*_D_ was set at 100 mV. The *V*_g_ of −26 V (pristine), −30 V (2 Å) and −32 V (10 Å) were used. I and II represent the wavelengths of 610 and 650 nm, respectively, corresponding to the split maxima of the inter-band transition of MoS_2_. III shows the wavelength of 705 nm, which appears after the deposition of CuPc.

To examine the variation in photo-current with wavelength more precisely, we measured the variation in *I*_d_ with a change in the wavelength of the incident light continuously using a computer controlled monochrometer, and the results are shown in Fig. 4.

We compared the results of the pristine channel and the CuPc-molecule deposited channels (2 Å and 10 Å in thickness). As already discussed in Fig. 3(b), the maximum photo-current of the molecule-deposited channel was 3.5 times higher than that of the pristine channel. Tracing the plot from the low energy side of the incident light (right-hand side of the *x*-axis), it can be seen that *I*_d_ showed a rapid increase at 680 nm. It showed two split maxima at 650 nm and 605 nm. According to the maxima, the drain current gradually decreased in the short-wavelength direction. Conversely, the plot for the sample with 2 Å CuPc molecules showed a gradual increase starting from 780 nm and split maxima similar to the pristine case. Interestingly, we observed the appearance of a new peak at the energy of ∼705 nm after the deposition of CuPc. For the sample with the deposition of 10 Å CuPc, it is apparent that the lower energy component increased in intensity. The total photo-current was about 1.2 times higher in intensity than that obtained after the deposition of 2 Å CuPc.

**Fig. 5:**
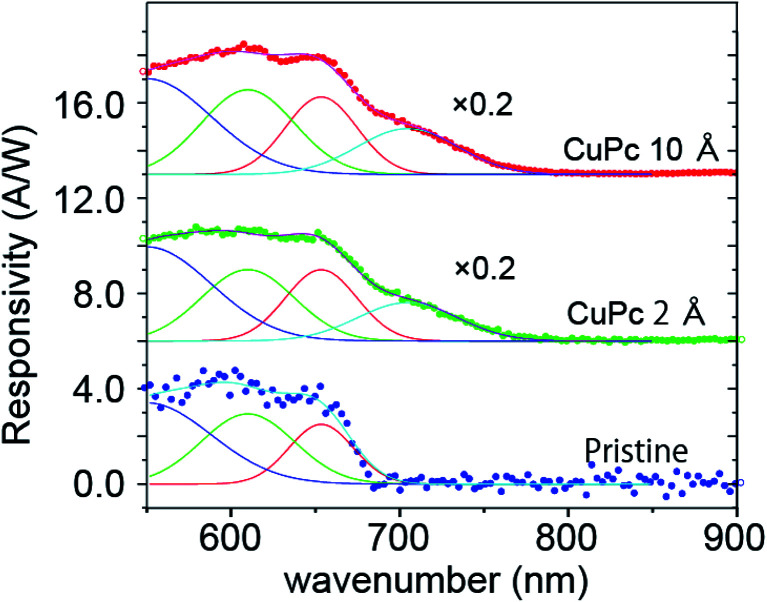
Calculated photo-responsivity as a function of the wavelength of the incident light for (a) pristine MoS_2_, (b) 2 Å CuPc and (c) 10 Å CuPc deposited cases. Experimental data points are plotted as thick solid line. The plot for the pristine surface is fitted with three Gaussian peaks with the center wavelength of approximately 550, 610, 650 nm, respectively, and an additional Gaussian peak centered at 705 nm was employed for the sample with CuPc.

We further analyzed the (Δ*I*_d_)_ph_*vs. λ* plot with the power of the incident light. We normalized the spectra with the intensity-*vs.*-wavelength property of the light (see Fig. 1(c)) and executed deconvolution into specific Gaussian components. The performance of the photo-conductance is often evaluated using photo-responsivity (*R*), which can be estimated using the formula *R* = *I*_Ph_/*P*_Light_, where *I*_Ph_ is the photo-current and *P*_Light_ is the power of the monochromatized light injected on the channel surface.^17,19^ The photo-responsivity in unit of ampere per watt (A W^−1^) is shown in Fig. 5.^20^

For the pristine channel, we used three Gaussian curves. The ones centered at 610 and 650 nm are split maxima and are visible in the raw experimental plot. The one centered at 550 nm is broad.

With the deposition of 2 Å CuPc, we observed an increase in the intensity in the longer wavelength side and we employed an additional peak centered at 705 nm for the fitting. When the coverage increased to 10 Å, we observed a further enhancement in the intensity of the peak at 705 nm, while the contribution from feature I and II was similar.

An increase in the drain current with the irradiation of the light for an FET with a pristine MoS_2_ channel was reported previously,^20,21^ where it was assumed that the increase in the drain current was initiated by the absorption of photons by MoS_2_.

The optical absorption spectrum of MoS_2_ has been examined in several reports, some of which intended to determine the inter-band transition energy.^22–24^ Splendiani *et al.* reported the photo-luminescence spectrum of single-layer MoS_2_. They reported two dominant peaks at 627 nm and 670 nm embedded in a continuous background for the wavelength region lower than 500 nm.^23^ These two enhanced peaks were assigned to the direct inter-band transition from the split valence bands to the CBM, which occurs at the *K*-point of the Brillouin zone. The appearance of the direct transition is due to the band structure change caused by the thinning of the MoS_2_ layer.^25^ The split in the valence band is attributed to the combinational effect of the spin–orbit coupling and the inter-layer coupling between the layers of MoS_2_.^22,25^ The origin of the continuous background below 500 nm in the MoS_2_ layer has been studied experimentally^26^ and theoretically,^27,28^ which was assigned to the parallel band or ‘band nesting’ effect and corresponding divergence in the joint density of states of MoS_2_.

**Fig. 6:**
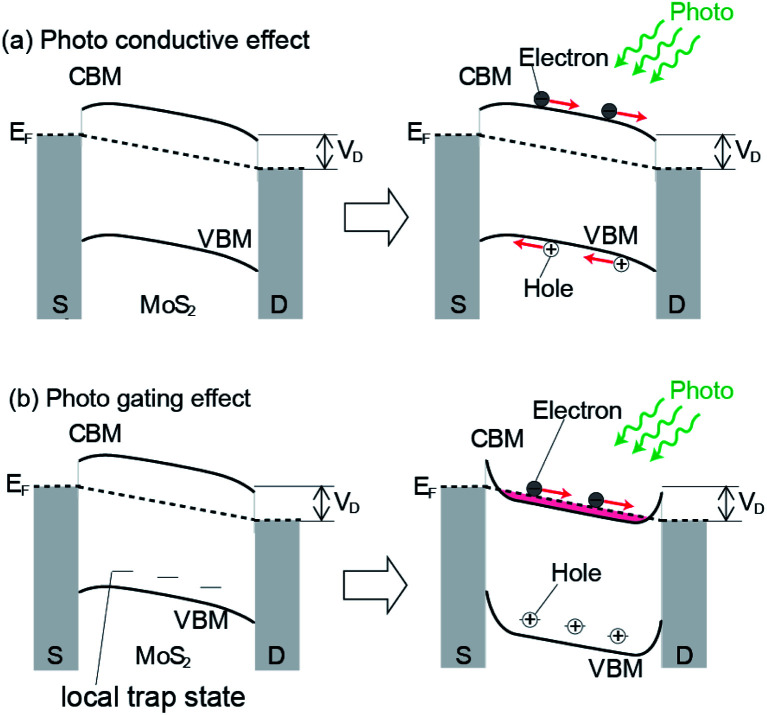
Schematic of the photo-conductive effect (a) and photo gating effect (b). Electrons and holes generated by incident light move toward the drain electrode and source electrode, respectively. The mobility of holes decreases due to the existence of the created hole in the local trap state after the electron is relaxed to the hole of the VBM.

Next, we determined how the absorption of light by MoS_2_ is converted into an increase in the photo-current. Two paths can be considered. The first model is the photoconductive mechanism, in which, the electron–hole pair created by light injection follows the electric field created by the drain voltage (illustrated in Fig. 6(a)) and is measured as the photo-current. The second is depicted in Fig. 6(b), which is the photo-gating effect. Initially, a hole is created by photoexcitation in the valence band, which is relaxed by the creation of holes in the local trap states existing in the gap. The holes in the trap states are immobile and provide an electric field, which works as an effective gate voltage to increase *I*_d_. In both cases, the photoexcitation efficiency determines the amount of photo-current. The existence of a trap state in an MoS_2_ FET was first demonstrated by Ayari *et al.* based on the *I*_d_–*V*_g_ measurement of the MoS_2_ FET.^29^

Based on these studies, Furchi and coworkers discussed that a considerable part of the drain-current increase is due to the photo-gating effect, where the trap state remains for a long time. According to the experimental results, they calculated the values of the subthreshold swing and photo-responsivity, which were estimated to be 2 V dec^−1^ and 6 A W^−1^, respectively. These values are much larger than that expected for a photo-conductive process of 0.06 V dec^−1^ and 0.06 A W^−1^, respectively, which they attributed to the photo-gating effect.^21^ A similar discussion was reported for MoS_2_ and it was concluded that photo-gating is the dominant process of this phenomenon.^20^

The plot of (Δ*I*_d_)_ph_*vs. λ* for the device with the pristine MoS_2_ channel in our experiment is consistent with the results of these optical studies, with a sharp increase at 680 nm and split maxima at 605 and 650 nm together with a broad background around 500 nm. Thus, we successfully divided the photo-current into the corresponding components.

The presence of the maximum value of the (Δ*I*_d_)_ph_–*V*_g_ curve shown in Fig. 3(b) can be understood by considering that the CBM starts to be filled when *V*_g_ exceeds (*V*_g_)_r_. Considering that the CBM is the final state of photoexcitation, the excitation probability decreases with an increase in the occupation of the CBM state. Below (*V*_g_)_r_, the excited electron is effectively transported as the drain current, which increases (Δ*I*_d_)_ph_ with *V*_g_.

Here, we discuss the case of CuPc deposition. The effect of the adsorption of CuPc on the electric properties of an MoS_2_ FET was first studied by Pak and coworkers.^17^ They found that the photoresponsivity increased significantly in the presence of CuPc molecules in the channel region when photons with an energy higher than the band gap are irradiated. This was attributed to the increase in the efficiency of the creation of electron–hole pairs in the presence of CuPc molecules.

The electronic states of the CuPc/MoS_2_ system were investigated using various optical techniques. Amsterdam *et al.* performed UV-vis absorption spectroscopy for single-layer MoS_2_, CuPc and a combination of the two.^30^ For the MoS_2_ surface, a split absorption peak located at 600–700 nm was observed, which is consistent with the above-mentioned report.^23^ After the deposition of CuPc, in addition to these two peaks, a new feature appeared at ∼710 nm. Combined with theoretical calculation, they made a model in which the HOMO level is located just above the VBM and in the band gap of MoS_2_. The observed absorption peak at ∼710 nm is attributed to the charge transfer (CT) excitation.

A similar system was investigated by Padgaonkar and coworkers, in which the pump-probe technique was employed using fast laser light.^31^ Light with a wavelength of 705 nm was used as the pump light to excite the CuPc HOMO state to the unoccupied state of CuPc. It was found that the excited electron transferred within a short timescale of 1 ns to MoS_2_, which was revealed using the probe light detecting the ground state bleach of MoS_2_. This fast transfer indicates that the molecule-induced state, in which the photo-excited electron is transferred, is strongly related to MoS_2_. The transfer of the hole from CuPc to MoS_2_ is not probable energetically, given that the HOMO level is higher than the VBM of MoS_2_.^17,30,32^

We believe that these previous studies can be combined to create a model in which a molecule-induced state (MIS) is assumed to be formed near the CBM level of MoS_2_. The energy separation between the HOMO and the MIS level corresponds to 705–710 nm. The HOMO state of CuPc is located in the energy gap of MoS_2_ and above the VBM. The MIS is strongly associated with the CBM band of MoS_2_, which enables fast electron transfer. The feature in the UV-vis absorption spectra at 710 nm has a relatively broad peak-width, which is larger than 100 mV, resulting from the strong coupling of these two states.

The strong coupling between the MIS and the MoS_2_ surface was also reported in a photo luminescence experiment. Nguyen *et al.* observed that the signal of the photo luminescence from CuPc was quenched by an MoS_2_ nanosheet, which shows electron transfer from the CuPc excited state to MoS_2_.^33^

**Fig. 7:**
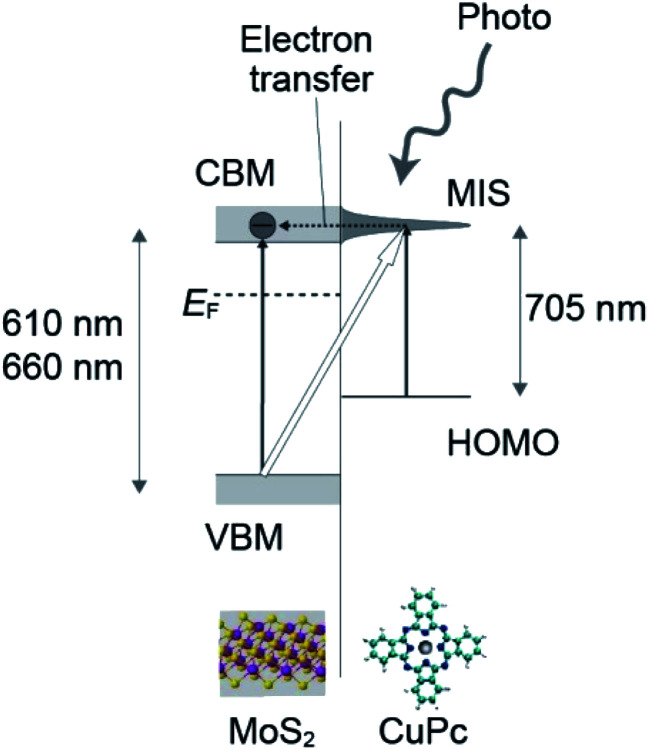
Schematic drawing of the excitation from the HOMO to unoccupied state in CuPc followed by fast electron transfer to the CBM of MoS_2_. The transferred electrons are detected as the drain current, which is characterized by the 705 nm feature in this work.

Considering all these previous works, the 705 nm feature in our work can be assigned to the excitation from the HOMO state to the MIS level, followed by fast electron transfer to the conduction band of MoS_2_. The transferred electrons were detected as the drain current. A schematic drawing of the process is shown in Fig. 7.

It should be mentioned that the energy positions of the MIS states, which should be detected as the HOMO–MIS excitation energy in our experimental set-up, can be used as the chemical fingerprints of various molecules. Actually, a previous report on the optical-absorption experiments executed for various Pc family molecules showed the differences in the peak positions and the relative peak intensities for the molecule-induced peaks.^30^ The same scenario should be applied to our detection method and the chemical difference of the adsorbates on the MoS_2_ channel should be detected in the drain current. This examination is currently underway.

In our current experiment, after the creation of an electron–hole pair in the CuPc layer by light injection, the electron transfers to the CBM band quickly and dissipates into the drain, which contributes to the photo-current. Conversely, the hole should remain at the excited site. This is due to the low mobility of the hole in the CuPc layer and the reduced possibility of electron–hole relaxation due to the disappearance of the electron. The hole works as an effective gate, similar to the case stated above as photo-gating. Both the photo-current and photo-gating should contribute to the significant increase in *I*_d_.

We should discuss why the photo-current peak at ∼600 nm increases with CuPc deposition at the same wavelength. In the previous work by Pak *et al.*,^17^ it was mentioned that the adsorption of light proceeds in a parallel manner by MoS_2_ and CuPc molecules. Using the model we discussed above, we can construct a more detail model. We consider that the MIS level is the key for this increase. The photo-adsorption proceeds not only by the VBM–CBM excitation in the MoS_2_ layer but also the VBM to MIS state. Considering that the energy of the MIS state is well aligned with that of the CBM, this excitation occurs at a similar energy with that of VBM–CBM excitation. Due to the strong coupling between the MIS and CBM state, the excitation from the VBM to MIS will occur with a high yield. In addition, the excited electrons are efficiently transferred to the MoS_2_ channel as the drain current. These properties contribute to the in the photo-current at 600 nm upon the adsorption of CuPc.

## Summary

We successfully enhanced the chemical recognition capability of a molecule sensor composed of an atomically thin MoS_2_ FET by injecting visible light in its channel. By injecting light, we found that *I*_d_ increased from the light-off state, where the difference between the light-on and off state is defined as (Δ*I*_d_)_ph_. We examined (Δ*I*_d_)_ph_*vs.* light wavelength (*λ*) by injecting a continuous spectrum of light. We executed experiments both in under UHV-measurement condition and N_2_ atmosphere-measurement condition. The (Δ*I*_d_)_ph_–*λ* plot for the pristine channel shows a clear onset and split-maxima, corresponding to the excitation from the valence band minimum (VBM) to the conduction band minimum (CBM), whose spectrum is similar to the previously reported photo-absorption spectrum of a single-layer MoS_2_ sample. The chemical sensitivity was demonstrated using the CuPc molecule. After the deposition of CuPc on the channel, we observed the appearance of a new peak in the (Δ*I*_d_)_ph_*vs. λ* spectrum, corresponding to the excitation from the HOMO state to the molecule-induced state (MIS) formed near the CBM of MoS_2_. We believe that this technique can be employed for molecular sensing with chemical recognition capability.

## Conflicts of interest

There are no conflicts to declare.

## Supplementary Material

RA-011-D1RA03698J-s001
