# Supplementary material for: Sensor behavior of MoS2 field-effect transistor with light injection toward chemical recognition
Source: RSC Adv. 2021 Aug 3;11(43):26509–15. doi: 10.1039/d1ra03698j (PMC9037302; doi:10.1039/d1ra03698j)

Electronic Supplementary Material (ESI) for RSC Advances.
This journal is © The Royal Society of Chemistry 2021

Supplementary Information

Sensor Behavior of MoS_2 Field Effect Transistor with UV/Vis Light Injection Toward Chemical Recognition

Md Iftekharul Alam[1], Tsuyoshi Takaoka[2], Hiroki Waizumi[1], Yudai Tanaka[1], Muhammad Shamim Al Mamun[1], Atsushi Ando[3] and Tadahiro Komeda[2]

[1]*Faculty of Science, Tohoku University, Japan*

[2]*IMRAM, Tohoku University, Japan*

[3]*National Institute of Advanced Industrial Science and Technology, 1-1-1 Umezono, Tsukuba, Ibaraki, 305-8568*

E-mail: Tsuyoshi Takaoka: tsuyoshi.takaoka.b1@tohoku.ac.jp, Tadahiro Komeda: tadahiro.komeda.a1@tohoku.ac.jp

S1. Process flow of FET device fabrication

Small piece of commercially available MoS_2 crystal was chosen and highly doped silicon (p++ Si) wafer with 300 nm SiO_2 thermal oxidation layer was used to transfer the desired flakes using mechanical exploitation process by scotch tape. Before MoS_2, transfer SiO_2/Si

(a)

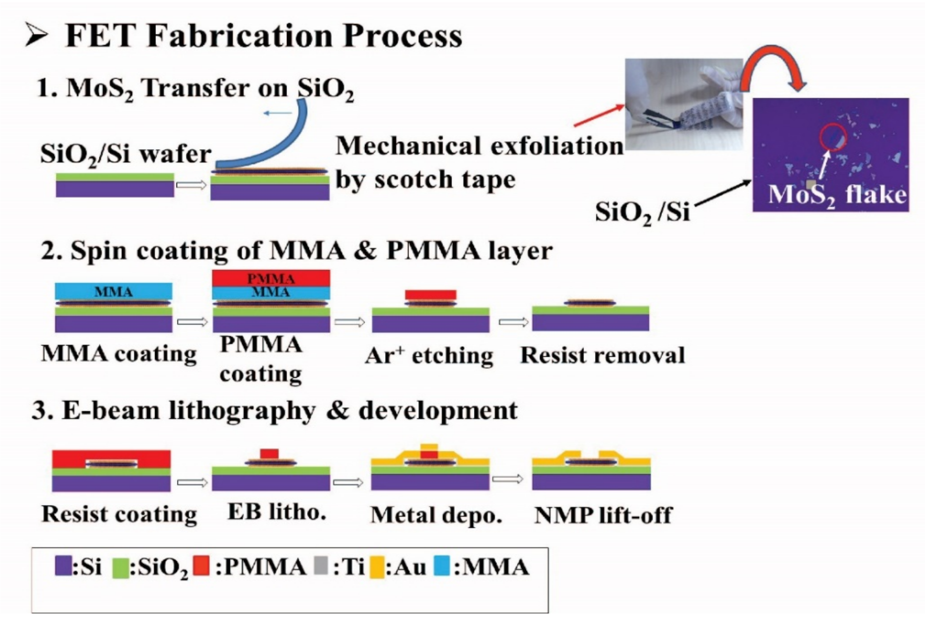

(b)

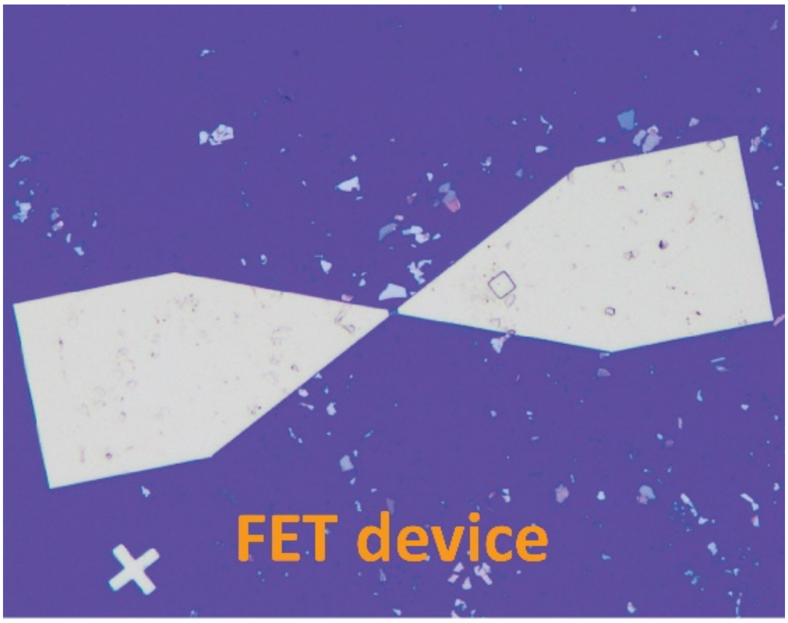

Figure S1: (a) Flow process of device fabrication with MoS_2 channel material. The length (L) and width (W) of selected MoS_2 flake are 16.05 μm and 3.28 μm, respectively. MoS_2 layer

number was estimated using optical contrast formula and that lies in between 5~6 (b) Optical image of fabricated MoS_2 FET device.

substrate was properly treated with ethyl alcohol, IPA, and DI water to remove unwanted adsorbates and finally treated with UV/O_3 to remove a variety of contaminants from surfaces, making suitable for MoS_2 transfer. Optical microscope was used to check the layer number by the optical contrast, using $C = (R_0-R)/R_0$ where R_0 and R represent the reflected light intensities from the air/(SiO_2 on Si) and air/MoS_2/SiO_2/Si system, which are wavelength dependent [S1]. Figure S1(a) demonstrates the overall process flow of MoS_2 device fabrication. The MoS_2 pasted SiO_2/Si substrate was prepared for a methyl methacrylate (MMA) and polymethyl methacrylate (PMMA) bilayer resist process, using spin coater. Afterwards, electron-beam lithography (EBL) was used to pattern source (S) and drain (D) electrodes on selected MoS_2 flakes with different channel length. Moreover, the S/D patterned substrate was then placed in spin coater with MMA and PMMA under a baking temperature at 180 ºC for 2 min for all steps. Source and drain (S/D) electrodes were created using electron beam lithography and 10nm Ti incorporated with 150 nm Au was deposited in an eb- evaporator. Source/drain(S/D) electrodes were visible after standard lift-off process in acetone. Finally, MoS_2 FET is appeared that has been shown in Figure S1 (b).

S2. Preparation of device and light source for vacuum measurement

The fabricated MoS_2 FET was then prepared for vacuum observation. Figure S2 (a) shows multiple steps of FET arrangement compatible under vacuum investigation that can be categorized as following. Firstly, a copper epoxy sheet was designed including source (S), drain (D) and gate (G) connection according to our FET device holder. Secondly, H21D conductive glue was used to fix the FET device on the middle of gate connection with proper procurement. Thirdly, source and drain connections were executed by using a wire bonder machine where aluminum wires with a diameter of 0.025 mm were used from the source and drain electrodes respectively and electrical properties were studied in air after inserting in device holder finally. Note that, transfer characteristics (I_{DS}-V_g) for both before and after wire were almost identical, was also observed. The chamber was next prepared to make it vacuum

by proper pumping followed by short baking for about 6 hours and the UHV pressure was recorded as 3×10^{-6} Pa. The device was later introduced to light after conforming the stable

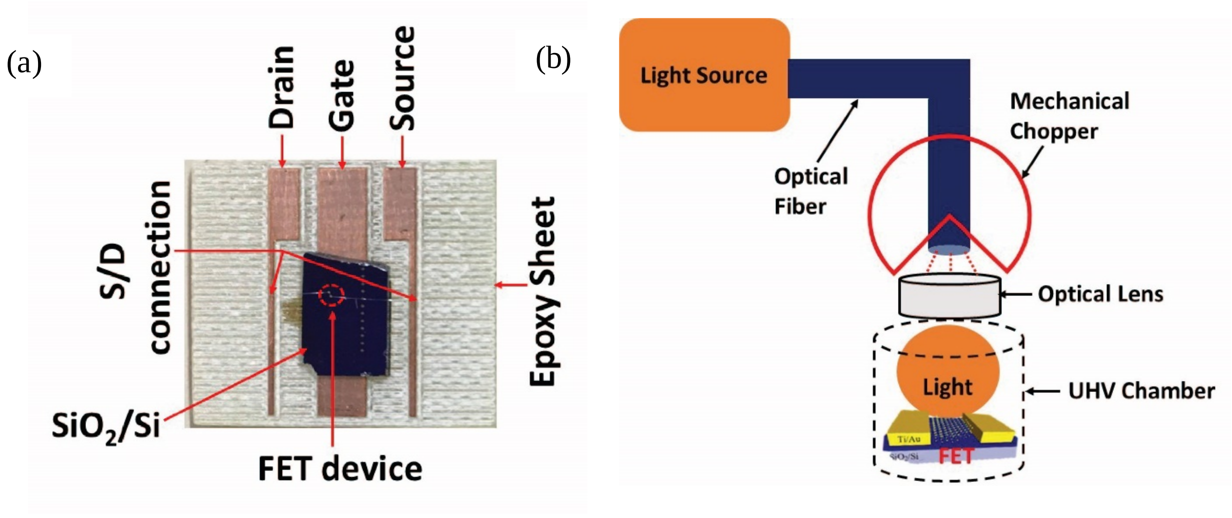

Figure S2: (a) An image of copper epoxy sheet including source, drain and gate connection where FET device was fixed on the top of gate connection. The source and drain connections were attached to respective positions using aluminum wires (b) Schematic of overall light injection procedures used in our studies.

I_{DS}-V_g characteristics under cooled vacuum chamber. The overall light injection procedure has been demonstrated in Figure S2 (b). Light was injected through a monochromatic light source by an optical fiber that was directed on to an optical lens. A mechanical chopper having a hole to introduce light, was attached in between optical fiber and optical lens. The light is injected through the hole with a 4s interval i.e. to complete the rotation of chopper to

S3. Control experiment of a sample without MoS_2 channel

There is a possibility that the feature characterized by the position of 710 nm is assigned to the absorption in the CuPc adlayer only without MoS_2 layers. It has been reported that CuPc film works as an FET after deposited on SiO_2 surface with source and drain electrodes. Then, we measured the electric properties of the CuPc film deposited on the SiO_2 surface with source and drain electrodes. The results are shown in figure S3. The figure shows time-dependent drain current plots during the injection of light for the wavelength of 600 nm for (a) CuPc(10 Å) /SiO_2 (blue line), and (b) CuPc(10 Å) /MoS_2/SiO2 (red line). The figure shows that the magnitude of the photocurrent for the CuPc(10 Å) /SiO_2 sample without MoS2 layers is below the detection limit.

In addition, we obtained XPS spectra to confirm the existence of CuPc on both CuPc(10 Å) /SiO_2 surface and CuPc(10 Å) /MoS_2/SiO_2 surface. Figure S3 (b) show the XPS

spectra in the range corresponding to Mo3d and Cu2p for (a) SiO2 surface, (b) CuPc(10Å) /SiO_2 (blue line), and (c) CuPc(10Å) /MoS_2/SiO_2 surface. As shown here, it is confirmed that CuPc exists on both CuPc(10Å) /SiO_2 surface and CuPc(10Å) /MoS_2/SiO2 surface. Therefore, we conclude that the fourth Gaussian peak at 710 nm shown in Figure 6(b), (c) appears from the CuPc adsorbed on MoS_2 surface, not from the CuPc without MoS_2 surface.

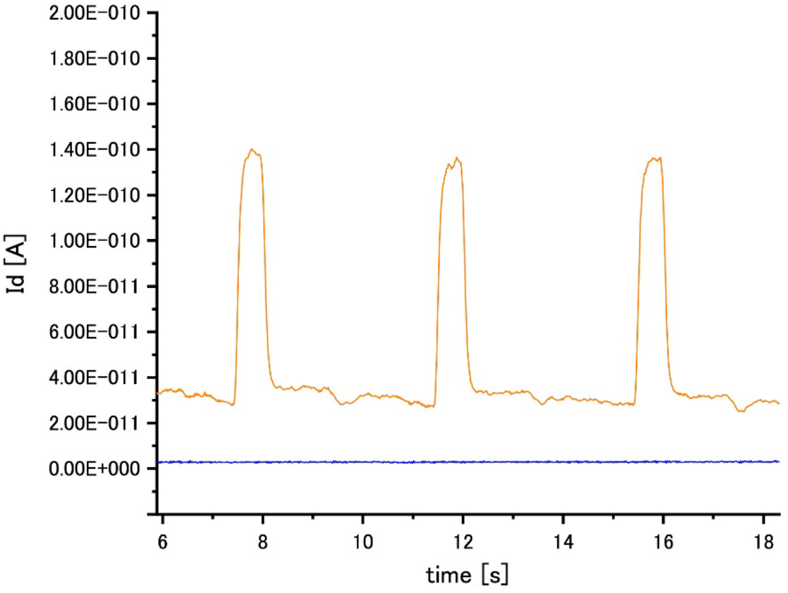

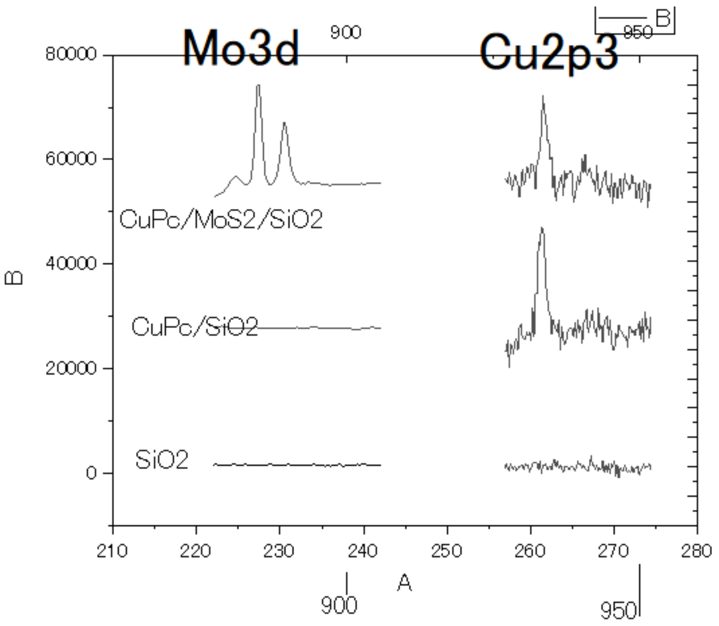

Supplement: RA-011-D1RA03698J-s001 [file RA-011-D1RA03698J-s001.pdf]
